# Genome-Wide Egg Hunt: Unhiding Candidate Genes for Egg Component Traits in Layers of an F_2_ Resource Population

**DOI:** 10.3390/ani15233391

**Published:** 2025-11-24

**Authors:** Natalia A. Volkova, Michael N. Romanov, Polina V. Larionova, Alan Yu. Dzhagaev, Ludmila A. Volkova, Alexander A. Sermyagin, Darren K. Griffin, Natalia A. Zinovieva

**Affiliations:** 1 L. K. Ernst Federal Research Center for Animal Husbandry, Dubrovitsy, Podolsk 142132, Moscow Oblast, Russia; natavolkova@inbox.ru (N.A.V.); volpolina@mail.ru (P.V.L.); alan_dz@inbox.ru (A.Y.D.); ludavolkova@inbox.ru (L.A.V.); n_zinovieva@mail.ru (N.A.Z.); 2 School of Natural Sciences, University of Kent, Canterbury CT2 7NJ, Kent, UK; 3 Animal Genomics and Bioresource Research Unit (AGB Research Unit), Faculty of Science, Kasetsart University, Chatuchak, Bangkok 10900, Thailand; 4 Russian Research Institute of Farm Animal Genetics and Breeding—Branch of the L. K. Ernst Federal Research Centre for Animal Husbandry, Pushkin, St. Petersburg 196625, Russia; alex_sermyagin85@mail.ru

**Keywords:** chicken (*Gallus gallus*), laying hens, genome-wide association study (GWAS), single nucleotide polymorphisms (SNPs), candidate genes, egg weight, yolk weight, albumen weight, eggshell weight, F_2_ resource population

## Abstract

Egg components, including weight of yolk, albumen, and eggshell, are economically important traits in poultry breeding and production, and we thus conducted a genome-wide association study (GWAS) for them. We used an F_2_ resource population of hens (*n* = 142) in different periods of laying, obtained by crossing breeds with contrasting phenotypes, and then genotyped them using the Illumina Chicken 60K iSelect BeadChip. Significant associations were found between 33 single nucleotide polymorphisms (SNPs) and yolk weight at 18–28 weeks of age (YW1). Eighty-seven SNPs were associated with thick albumen weight at 18–28 (TAW1) and 29–42 (TAW2) weeks of age. Four SNPs were associated with eggshell weight at 18–28 weeks of age (ESW1). Fifty-three candidate genes were identified in the positions of these SNPs, and seven prioritized candidate genes (PGCs) were revealed in regions where 2–4 SNPs associated with the studied traits were localized. These were as follows: *SYTL5* (YW1, TAW1), *FRY* (TAW1), *GABRG3* (YW1, TAW1), *ALDH1A3* (YW1), *VCL* (YW1), *HYDIN* (YW1), and *TIMP4* (TAW1). Allelic variants at the *ALDH1A3*, *VCL*, *HYDIN*, *FRY,* and *TIMP4* loci were associated with higher YW1 and TAW1. These SNPs and PGCs are potential genetic markers for characterizing egg weight parameters and their components in chicken breeding to achieve egg production improvements.

## Simple Summary

Certain features in eggs (including the weight of the yolk, albumen, and eggshell) are important economically for poultry breeding and production. This study aimed to establish if there are genes (and, more specifically, variants of genes) that are associated with these traits. To this end, we scanned the genomes of 142 hens phenotyped in different periods of laying; these hens had previously been obtained by crossing breeds with contrasting characteristics. We found a total of 33 gene variants that were associated with yolk weight at 18–28 weeks of age (we called these “YW1”). We found 87 that were associated with thick albumen weight at 18–28 weeks of age (TAW1) and 29–42 weeks of age (TAW2). Finally, four variants were associated with eggshell weight at 18–28 weeks of age (ESW1). These 124 variants were in 53 genes, of which we prioritized 7 genes on the basis that at least 2 variants were found in them. These genes, and the variants that they contain, are potential genetic markers for describing egg weight parameters and their components for the breeding of chickens and possibly other poultry. Using these molecular tools, egg production can be improved significantly through genetic selection.

## 1. Introduction

Modern industrial poultry farming is one of the most rapidly developing branches of the agricultural sector [1,2,3,4]. It is associated with both the creation and use of highly productive industrial crosses of egg- and meat-type poultry [5,6,7,8,9], and with high consumer demand for poultry products, i.e., meat and eggs [10,11,12]. A significant share of these products are table chicken eggs—a valuable food component sold and consumed directly for cooking and used in processed foods such as baby food [13,14,15]. The qualitative characteristics and nutritional value eggs are determined by the biochemical composition and properties of its main components, i.e., yolk, egg white (albumen) and eggshell [16,17,18,19,20,21]. The yolk contains fats, proteins, fatty acids, and macro- and microelements that have a significant impact on the taste of the egg and its nutritional and energy value. These are also required for the full development of the embryo [22,23,24,25,26,27]. Egg white serves as a source of protein for skeletal muscle growth [18]. This component is also important for embryo growth and its protection from bacterial contamination during incubation [28,29,30]. The shell performs a protective function, shielding the contents, including developing embryos, from external factors. It helps prevent water loss and the penetration of microorganisms during collection, transportation, storage, and incubation [31,32,33,34,35,36,37].

Egg weight (EW) is an important selection trait that traditionally receives significant attention in both egg-type and meat-type poultry industries [10,38,39]. The weight of commercial eggs determines their category and price for consumer sale as a food product. In addition, the weight of a hatching egg is, to a certain extent, related to the development of the embryo; for instance, the influence of EW and female age on the reproductive egg quality, embryo development, and hatchability of chicks has been well-described [40,41]. The egg is a closed system for the growth and development of the embryo outside the female’s body, thus the completeness of the poultry embryogenesis depends on the nutritional value and energy value of the egg [42,43,44,45,46,47]. Many studies demonstrated that chickens hatched from larger eggs are characterized by higher body weight (BW) and growth rate compared to chickens obtained from smaller eggs [48,49,50,51,52].

EW is determined by the combined mass of its main components, i.e., yolk, albumen, and shell. An important indicator characterizing both the nutritional value of a table egg and the quality of a hatching egg is the ratio of yolk to albumen [13,39,53,54,55]. Intensive selection of chickens for high egg production and efficient feed conversion has altered the ratio of yolk to albumen. A decrease in the proportion of yolk in chicken eggs of highly productive egg lines and crosses has been previously noted compared to purebred poultry that, consequently, leads to a reduced nutritional value [56]. In this respect, the weights of individual egg components are also individually important selection traits that should be taken into account when attempting to improve egg quality in industrial poultry farming conditions [57,58,59,60,61,62,63].

EW (and that of its components) depends on several factors, including genotype [64,65,66,67,68], feeding [34,69,70,71], housing conditions [34,72,73], age of layers [31,39,74], and egg storage [75,76,77]. In terms of the physiological status of a hen, EW correlates with the egg-laying period and the age of the hen [78,79,80]. The minimum values of EW are observed at the beginning of egg laying, reaching maximum values in later periods of egg laying, usually at the age of laying hens over 52 weeks [80,81]. An increase in EW is due to the weight elevation of its main components, i.e., albumen, yolk, and shell. At the same time, the growth in the weight parameters of the egg occurs, to a greater extent, due to the increase in egg white weight (EWW) [48,82]. Yolk weight (YW) also positively correlates with EW [83] and is the second-most important component (after protein) influencing weight parameters [48,82].

The genetic basis of EW and its components has been confirmed by many studies [64,84,85,86,87,88,89]. The intensity of egg poultry farming, as well as the profitability and competitiveness of this industry in modern agricultural production conditions, are facilitated by the use of scientific approaches. This includes the targeted selection of layers and the formation of breeding and productive flocks to leverage the use of genetic, genomic, and biological technologies [90,91,92,93,94,95]. The success of introducing these technologies into poultry industry practice depends primarily on the information about genetic markers for selectively significant traits and quantitative trait loci (QTLs) [96,97,98,99,100,101]. Clarification and updating of this information is inextricably linked with the study of molecular genetic mechanisms that determine the phenotypic variation in economically important traits and QTLs that are crucial for enhancing the efficiency of agriculture and increasing the production of competitive products [102,103,104,105,106].

The advent of the genomic era has opened up broad opportunities for the development of high-density single nucleotide polymorphism (SNP) arrays and other high throughput technologies. This allows for the implementation of genome-wide association studies (GWASs) [107,108,109,110,111]. GWASs increasingly play an important role in identifying previously undetected genetic associations of SNPs and candidate genes with important phenotypic traits and QTLs in chickens. These include egg quality indicators and the use of F_2_ resource populations [64,112,113,114,115]. In our earlier studies, we analyzed potential genes and selective signatures in parental lines of the layer Russian White (RW) and meat-type Cornish White (CW) breeds subject to strong divergent selection pressure for egg production [116]. SNPs and prioritized candidate genes (PCGs) associated with egg production traits were identified in the F_2_ resource population of chickens produced by crossing the same two breeds [117,118].

The aim of this study was to extend these previous investigations and focus on SNP detection and identification of candidate genes associated with egg component weights at different periods of egg production. These specifically included YW, EWW, and weights of thick albumen (TAW) and eggshell (ESW) at three age periods: 18–28, 29–41, and 42–52 weeks. In line with this aim, a GWAS for the weight parameters of the main egg components were conducted in F_2_ resource population hens obtained from a cross between the RW (with higher egg performance) and CW (with lower egg production) breeds using genome-wide genotyping data.

## 2. Materials and Methods

### 2.1. Experimental Birds and Performance Data Collection

The two original breeds of chickens were reared at the L. K. Ernst Federal Research Centre for Animal Husbandry (LKEFRCAH; Dubrovitsy, Russia) after hatching from eggs acquired from the Russian Research Institute of Farm Animal Genetics and Breeding (Pushkin, Russia). The LKEFRCAH facility was used to produce and raise the F_2_ resource population chickens. The latter were obtained using two contrasting, divergently selected breeds, RW and CW, and the F_2_ resource population development procedure was implemented as described elsewhere (e.g., [112,115,118,119,120]). In accordance with this procedure, RW was chosen because it is a layer breed and is characterized by higher egg production (up to 240 eggs per year). This breed was developed on the basis of White Leghorns crossed with local Russian chickens [121,122,123]. CW is a meat breed and is used as one of the parent stocks for producing highly productive meat crosses of broilers [116,124,125]. The performance of layers in this breed is much lower and reaches 120 eggs per year.

In the first stage of the F_2_ resource population creation, unrelated CW hens and RW roosters were selected. Four F_0_ population groups (F0_1, F0_2, F0_3, and F0_4) were formed, each of which consisted of one RW male and five CW females. From each group, F_1_ offspring were obtained and were used to produce the F_2_ population. For this purpose, F_1_ population groups were set up; each group included one F_1_ male and three F_1_ females that were not closely related. From each group, 60–80 F_2_ hens were obtained. The generated F_2_ progenies were employed as a model resource population for further molecular genetic studies to hunt for SNPs associated with the weight parameters of the main egg components in chickens. A total of 520 F_2_ individuals were raised, including 238 females. To conduct the GWAS, a sample of the F_2_ resource population (*n* = 142 females) was formed by taking into account the number of eggs laid from the age at first egg to the age of 28 weeks and the mean EW for this period. From each F_1_ sire, F_2_ descendants (layers) with contrasting (higher and lower) indicators of egg performance and EW were chosen in equal proportions for the GWAS analyses.

In terms of appropriate technological maintenance conditions as described elsewhere [126,127,128,129], F_1_ and F_2_ chickens were raised in brooders until the age of three weeks and then transferred to floor housing. For subsequent individual recording of egg productivity and egg quality, 17-week-old females were transferred to individual cage batteries. During the entire growing period, the birds had ad libitum access to feed and fresh water. Sufficient supply ventilation was provided in the rooms, ensuring the absence of dampness, drafts and gas pollution, while normal lighting was applied according to the age of the birds.

### 2.2. Phenotypic Characteristics and Their Analyses

The 142 F_2_ females of the resource population were phenotyped for the weight parameters of the egg and its components in three age periods: 18–28, 28–41, and 42–52 weeks. The selection of these age periods for assessing egg parameters was based on the physiology of egg laying and an analysis of age-related EW dynamics in the study population. This allowed us to identify the initial period of egg laying, the period of peak and intensive egg production, and the period of productivity decline. Eighteen weeks of age was defined as the initial age for recording and assessing the weight parameters of egg components in the hens of the study population, as this age is associated with the onset of egg production in hens.

Correspondingly, the following phenotypic indicators were assessed: EW at the age of 18–28 (EW1), 29–41 (EW2), and 42–52 (EW3) weeks; YW at the age of 18–28 (YW1), 29–41 (YW2), and 42–52 (YW3) weeks; EWW at the age of 18–28 (EWW1), 29–41 (EWW2), and 42–52 (EWW3) weeks; TAW at the age of 18–28 (TAW1), 29–41 (TAW2), and 42–52 (TAW3) weeks; and ESW at the age of 18–28 (ESW1), 29–41 (ESW2), and 42–52 (ESW3) weeks.

These indicators were recorded individually for each female. Eggs were assessed within 24 h after laying. Eggs and their components (yolk, egg white, thick albumen, and shell) were weighed on electronic scales with an error of 0.001 g. As a result, all laid eggs from each layer of the studied population were recorded and assessed daily from the age at first egg until the age of 52 weeks. Based on these experimental data, the mean values of EW, YW, EWW, TAW, and ESW were calculated for each layer in three age periods: 18–28, 28–41, and 42–52 weeks. Finally, the overall mean values EW, YW, EWW, TAW, and ESW in each of the considered egg-laying periods were used for the GWAS.

To assess the differences in the produced phenotypic values, i.e., the quantitative traits of eggs from the F_2_ resource population hens, a statistical approach based on the generalized linear models (GLMs) method was used as implemented in the STATISTICA 10 program (StatSoft, Inc./TIBCO, Palo Alto, CA, USA).

The GLM equation was as follows:

(1) $$y_{kij}=\;\mu +{{Age\_week}_{k}+PG}_{i}+{Hatch}_{j}+e_{kij},$$

where *y* is the value of the resultant trait of an individual in the *k*th age group according to the egg-laying period, the *i*th parent family group of the resource population, and the *j*th hatching batch during incubation; *µ* is the mean value of the trait within each group of traits in the studied sample of the chicken resource population; *Age_week* is the fixed effect of the *k*th age group by the egg-laying periods (i.e., three egg-laying periods); *PG* is the fixed effect of the *i*th parent family group of the F_2_ resource population (i.e., five F_1_ parent groups used to obtain the F_2_ population); *Hatch* is the fixed effect of the *j*th hatching batch during incubation (i.e., 15 hatches of the F_2_ population); and *e* is the residual (undistributed) variance of the equation model.

### 2.3. Sampling and DNA Extraction

DNA was isolated from feather pulp using a commercial Syntol kit for DNA extraction from animal tissue (Syntol, Moscow, Russia). A Qubit 3.0 fluorimeter (Thermo Fisher Scientific, Wilmington, DE, USA) was used to determine the concentration of the isolated DNA. The purity of the obtained DNA was assessed based on the OD260/280 ratio using a NanoDrop-2000 spectrophotometer (Thermo Fisher Scientific).

### 2.4. Genotyping and Quality Control of SNPs

Whole-genome genotyping of 142 chickens was carried out using the Illumina (San Diego, CA, USA) Chicken iSelect BeadChip containing 60K SNPs. Quality control and filtering of genotyping data for each sample and each SNP were performed in the R-4.0 software environment [130,131] using the PLINK 1.9 software package [132,133] and applying the following filters available in the program: --mind 0.10 (excludes samples with more than 10% of the genotypes for SNPs from the analysis), --geno 0.10 (excludes SNPs that are missing in more than 10% of samples), --maf 0.03 (excludes SNPs in which the minor allele occurs with a frequency of less than 3%), and --hwe 1e-6 (excludes SNPs that deviate significantly from Hardy–Weinberg equilibrium, *p* < 1.06 × 10^−6^). After pruning, 47,432 SNPs were utilized for further analysis.

### 2.5. Principal Component Analysis

Principal component analysis (PCA; [134]) was performed based on the variance-standardized correlation matrix and using PLINK 1.9. Data files were prepared in R-4.0 [130,131]. Data visualization was performed using the R package ggplot2 (version 3.5.2; [135,136]).

### 2.6. GWAS Scan

The search for SNP associations with the studied indicators of the egg component weight parameters in F_2_ chickens of the resource population was carried out using regression analysis in PLINK 1.9. Multiple linear regression [137,138,139] was employed for estimating quantitative traits (i.e., weights of individual egg components). The linear regression model in PLINK 1.9 for the considered quantitative traits of egg components was represented as follows:

(2) $$Y=\beta_{0}+\beta_{1}\times {SNP}_{1}+\beta_{2}\times {SNP}_{2}+\beta_{3}\times {SNP}_{3}+\ldots +\beta_{k}\times {SNP}_{m}+\varepsilon$$

where *Y* is a quantitative trait of egg components being considered; *β*_0_, *β*_1_, *β*_2_, *β*_3_, and *β_k_* are the regression coefficients for *k*-values of SNPs, with *β*_0_ as a free term; *SNP*_1_, *SNP*_2_, *SNP*_3_, and *SNP_m_* are the genotypes by marker for each *m*th SNP taken into account in the analysis; and *ε* is the residual error.

The assumptions of the linear regression model included the following: the linearity of the association between genotype and phenotype, independence of observations (via control for population stratification using PCA), constant error variance, and normal error distribution. Population stratification was performed for four principal component vectors.

The significance of the identified associations and the definition of significant regions in the chicken genome were assessed based on the Bonferroni null hypothesis test at a threshold of *p* < 1.06 × 10^−6^. Data visualization was performed in the qqman package (version 0.1.9; [140,141]).

The candidate genes in the regions of the identified SNPs, including the SNP position and 0.2 Mb flanks on both sides, were searched according to the chicken (*Gallus gallus*; GGA) reference genome assembly GRCg6a [142] and Genome Data Viewer in the NCBI chicken databases [143]. To obtain extended information on the identified SNPs, the Ensembl Genes release 106 database and the Ensembl BioMart data mining tool were used [144] as described elsewhere [116]. Functional annotation and gene ontology (GO) term enrichment analysis for the identified candidate genes were performed using the Ensembl BioMart data mining tool and the Database for Annotation, Visualization, and Integrated Discovery (DAVID Knowledgebase; version DAVID 2021 (December 2021), v2023q4, updated quarterly) [145,146]. Using ggplot2, data visualization box plots were generated for dependences between selected PCG genotypes (allelic variants) and egg component weight values.

## 3. Results

### 3.1. Analyses of Phenotypic Data on EW Parameters and Population Stratification

Table 1 presents descriptive statistics characterizing the distribution of values established for the weight indicators of eggs and their components in F_2_ hens of the resource population. The values of the studied traits varied depending on the age of the laying hens, reaching maximum values at the age of 42–52 weeks. In particular, the mean EW of the experimental hens in this age period reached 57.8 ± 0.53 g, which was 34% (*p* < 0.01) and 14% (*p* < 0.01) higher than the values of this trait established in the first (18–28 weeks) and second (29–41 weeks) periods of egg laying, respectively.

Table 2 shows the results of the GLM analysis used to estimate the significant effects of fixed factors (Fisher’s *F*-statistics) included in the model on the variability of phenotypic indicators, i.e., the analysis of variance (ANOVA) main effects.

The results of phenotypic variation decomposition for the studied traits of egg components in the examined sample hens (Table 2) showed, as expected, the most significant influence of the egg laying age factor (*p* < 0.001), with its highest effect for EW (*F* = 237.9) and YW (*F* = 245.5) and the lowest one for TAW (*F* = 40.3). The parent group factor had the highest significant effect on TAW variation in the bird age dynamics of egg laying (*p* < 0.046), while being insignificant, although on the verge of the trend threshold, for EWW. As for the chick hatching batch during incubation, this factor was insignificant, with a trend at *p* < 0.10 for EWW (*p* = 0.077) and EW (*p* = 0.067). These data may suggest the potential influence of environmental (technological) factors on the experimental output, along with physiological status of hens (i.e., age of the birds) and the genetic group of parental effects.

Judging from the above, the appropriate calculations were made for the studied weight characteristics of chicken eggs based on estimates generated by the least squares method (LSM) using the GLM procedure. This allowed us to understand the probable differences when leveling out fixed factors affecting the variation in egg quality indicators. The results of calculating the LSM-assisted estimates for the quantitative parameters of the egg components in F_2_ resource population hens are presented in Table 3. This data took into account the age period of egg laying using the GLM approach and was based on the main effects model (without interaction).

The generated LSM-based estimates are on the whole consistent with the phenotypic values in Table 1. However, they have, at the same time, a more accurate (aligned) expression of dependences for the examined sample of hens in relation to the age of egg laying, the parent family group of the F_2_ resource population, and the chicken hatching batch. These fixed factors were used for the further GWAS analysis to improve the accuracy of significant SNP identification.

**Figure 1:**
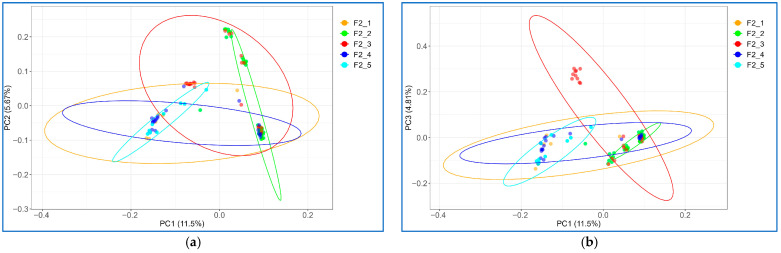
Principal component analysis (PCA) showing the F_2_ chicken resource population stratification (**a**) in the plane of the first principal component 1 (PC1; *X*-axis) and second (PC2; *Y*-axis) components, and (**b**) in the plane of PC1 (*X*-axis) and third component (PC3; *Y*-axis). Individuals from different groups are indicated by different colors.

To control for stratification of the resource population during GWAS, PCA was performed. PCA output demonstrated the distribution of the studied F_2_ resource population into several clusters depending on the F_1_ males used to obtain the F_2_ offspring (i.e., groups F2_1, F2_2, F2_3, F2_4, and F2_5). In the projections of the first three principal components, i.e., PC1 vs. PC2 and PC1 vs. PC3, the presence of several overlapping groups was noted (Figure 1a,b). In the subsequent GWAS analysis to search for significant associations of the studied traits, the first four principal components were taken into account, reflecting the structure of the F_2_ resource population.

### 3.2. GWAS Analysis

**Figure 2:**
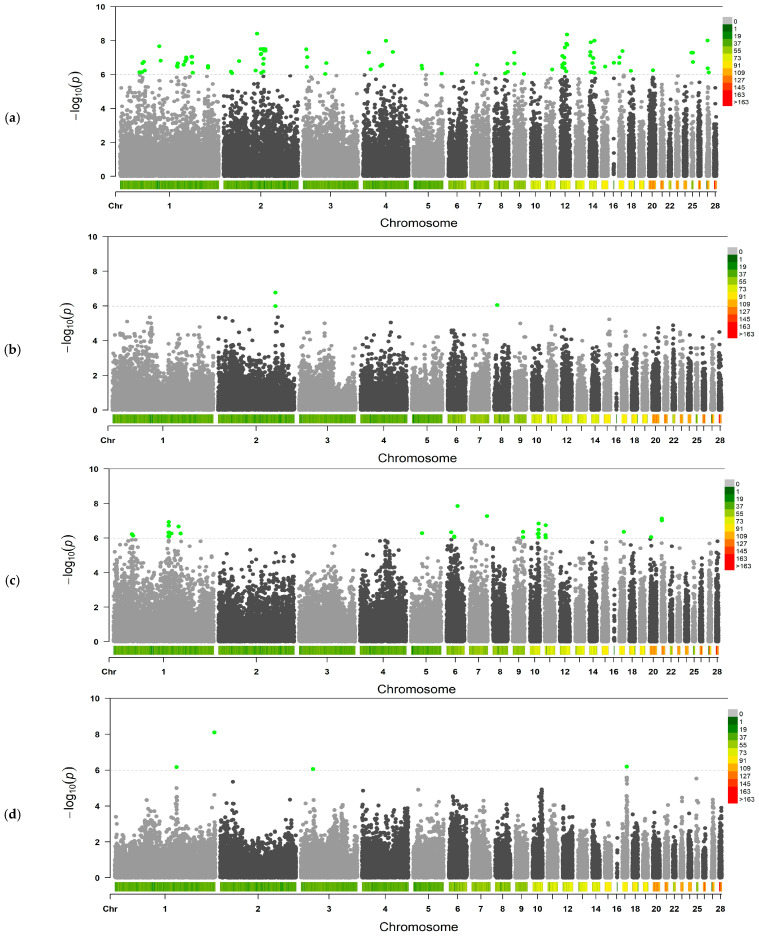
Manhattan plots based on the GWAS outcome for the studied egg quality traits in F_2_ hens from the resource population: (**a**) TAW1, thick albumen weight at 18–28 weeks of age, (**b**) TAW2, same at 29–41 weeks of age, (**c**) YW1, yolk weight at 18–28 weeks of age, and (**d**) ESW1, eggshell weight at 18–28 weeks of age. Manhattan plots imply distribution of single nucleotide variants in hens’ chromosomes at the significance level (−log_10_(*p*)) according to the expected Bonferroni probability value of *p* < 1.06 × 10^−6^ (dotted line) for the traits. Dots are color-coded only to visualize significant values. SNPs that have significant associations with the studied traits at the level of established reliability values from *p* < 1.06 × 10^–6^ are highlighted in green. The diagram along the *x*-axis shows the density of established SNPs on individual chromosomes.

The obtained phenotypic data on the weight parameters of the main egg components (i.e., yolk, egg white, and shell) in the F_2_ resource population females were used for the GWAS analysis. Of the 12 indicators taken into account (four traits by three age periods), only 4 were found to have significant SNPs at the established Bonferroni significance threshold (*p* < 1.06 × 10^−6^). The results, reflecting significant associations of the studied traits, are presented in Figure 2.

Table 4 presents data on the number of identified significant SNPs and their distribution on chromosomes, taking into account each specifically studied indicator of the egg components in F_2_ resource population hens.

The conducted GWAS of egg component weight parameters in hens of the F_2_ resource population revealed 122 SNPs associated with the studied traits in different egg lay periods (Supplementary Table S1). In particular, in the first considered period of egg laying (age of 18–28 weeks), significant SNPs associated with TAW1, YW1, and ESW1 were identified in the amount of 85, 33, and 4 SNPs, respectively. In the second period (age of 29–41 weeks), two SNPs associated with TAW2 were discovered. The found SNPs were established on 21 of 28 considered autosomes. A significant proportion of these SNPs were localized on GGA1 (29 SNPs), GGA2 (14 SNPs), GGA12 (eight SNPs), and GGA14 (nine SNPs).

Notably, we detected two SNPs, i.e., Gga_rs13939653 on GGA1 and Gga_rs14680477 on GGA9, that were significantly associated with the two studied traits, i.e., YW1 and TAW1.

### 3.3. Identification of Candidate Genes

Following the determination of significant SNPs associated with the studied weight parameters of the main egg components, we annotated the corresponding candidate genes associated with these traits. In the regions of identified SNPs (i.e., SNP position ± 0.2 Mb), 319 genes were found, including 53 candidate genes in SNP positions. These 17 genes were associated with YW1, 33 with TAW1, 2 genes with TAW2, and 3 genes with ESW1 (Supplementary Table S1). Here, seven PCGs were established, in the regions of which 2 to 4 SNPs associated with the studied traits were colocalized as follows: *SYTL5* (synaptotagmin like 5; YW1, TAW1), *FRY* (FRY microtubule binding protein; TAW1), *ALDH1A3* (aldehyde dehydrogenase 1 family member A3; YW1), *GABRG3* (gamma-aminobutyric acid type A receptor gamma3 subunit; YW1, TAW1), *VCL* (vinculin; YW1), *HYDIN* (HYDIN, axonemal central pair apparatus protein; YW1), and *TIMP4* (TIMP metallopeptidase inhibitor; TAW1).

The list of these PCGs associated with the weight parameters of the main egg components in F_2_ resource population hens is presented in Table 5.

Taken together, the functionally annotated candidate genes formed 19 functional clusters based on the GO term enrichment score, including 7 clusters with enrichment scores higher than 1.15 and 5 clusters with enrichment scores of more than 1.4 (Supplementary Table S2). These clusters included genes associated with ubiquitin-dependent protein catabolic process, ubiquitin protein ligase activity, glycoprotein, protein tyrosine phosphatase activity, protein phosphatase, lipid degradation, lipid metabolism, lipid catabolic process, and lipid metabolic process. Remarkably, one of the significant GO terms (FDR = 0.028) was related to KW-0325~Glycoprotein and comprised a total of 23 candidate genes.

### 3.4. Allelic Variants of Genes Determining EW Trait Manifestation

Allelic variants of the *HYDIN*, *VCL*, *ALDH1A3*, *TIMP4,* and *FRY* genes were examined in the positions of the SNPs identified in this study and associated with the weight characteristics of the egg components in F_2_ resource population layers.

**Figure 3:**
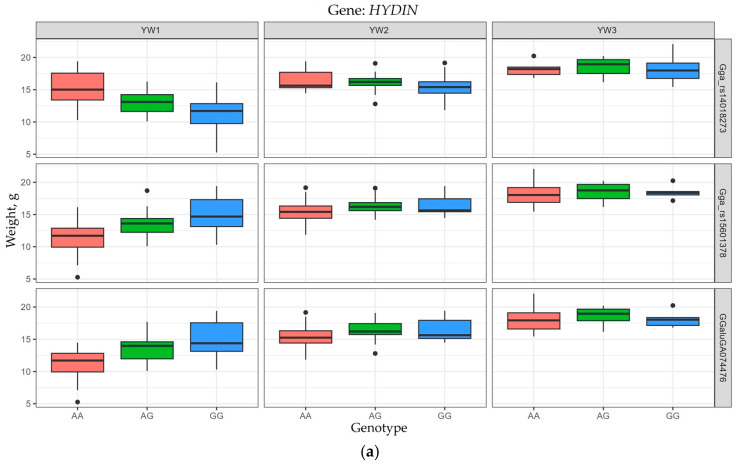

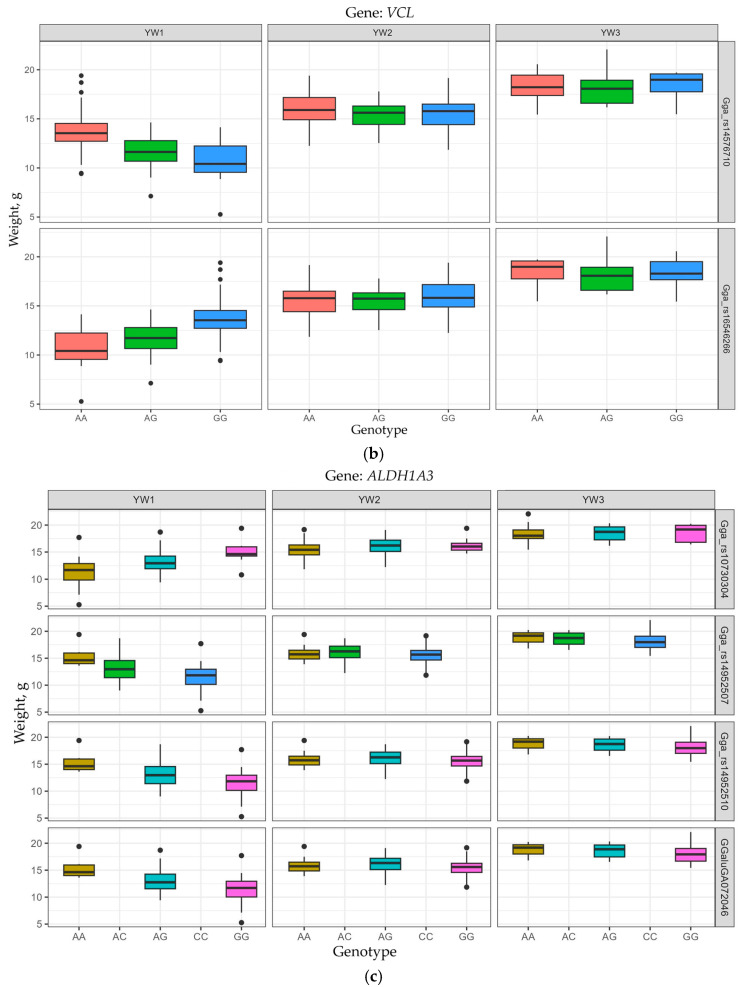
Data visualization box plots (using ggplot2) for yolk weight (YW) of eggs in F_2_ hens of the resource population depending on the genotypes for the genes *HYDIN* (**a**), *VCL* (**b**), and *ALDH1A3* (**c**). YW1, YW2, and YW3 conform to YW values at the ages of 18–28, 29–41, and 42–52 weeks, respectively.

The frequencies of the AA, AG, and GG genotypes at three *HYDIN* loci detected, i.e., Gga_rs15601378 (11:1592394), Gga_rs14018273 (11:1614369), and GGaluGA074476 (11:1626527), were 0.640, 0.271, 0.087; 0.125, 0.269, 0.605; 0.625, 0.296, and 0.115, respectively. The AA genotype at the Gga_rs14018273 locus and the GG genotype at the Gga_rs15601378 and GGa-luGA074476 loci significantly correlated with a higher YW1 value (*p* < 0.001). A similar significant trend (*p* < 0.001) was observed for YW2 (Figure 3a and Supplementary Table S3).

For the *VCL* gene, the frequencies of the AA, AG, and GG genotypes at the Gga_rs16546266 (6:16277262) and Gga_rs14576710 (6:16283091) loci reached 0.185, 0.369, 0.447; 0.433, 0.385, 0.183, respectively. The AA genotype at the Gga_rs14576710 locus and the GG genotype at the Gga_rs16546266 locus significantly contributed to a higher YW1 value (*p* < 0.001), with this significant trend (*p* < 0.001) continuing for YW2 (Figure 3b and Supplementary Table S3).

The following distribution of genotype frequencies was observed for the *ALDH1A3* gene in the studied chicken population: the frequencies of the AA, AG, and GG genotypes at the Gga_rs14952510 (10:17878899) and GGaluGA072046 (10:17898445) loci were 0.096, 0.230, 0.673 and 0.106, 0.298, 0.596, respectively, while those at the Gga_rs10730304 locus (10:17910258) were 0.573, 0.340 and 0.087. For the genotypes AA, AC, and CC at the Gga_rs14952507 locus (10:17878734), these were 0.096, 0.231, and 0.673. Genotype AA at the Gga_rs14952510, GGa-luGA072046, and Gga_rs14952507 loci and genotype GG at the Gga_rs10730304 locus significantly (*p* < 0.001) correlated with a higher YW1 values (Figure 3c and Supplementary Table S3).

**Figure 4:**
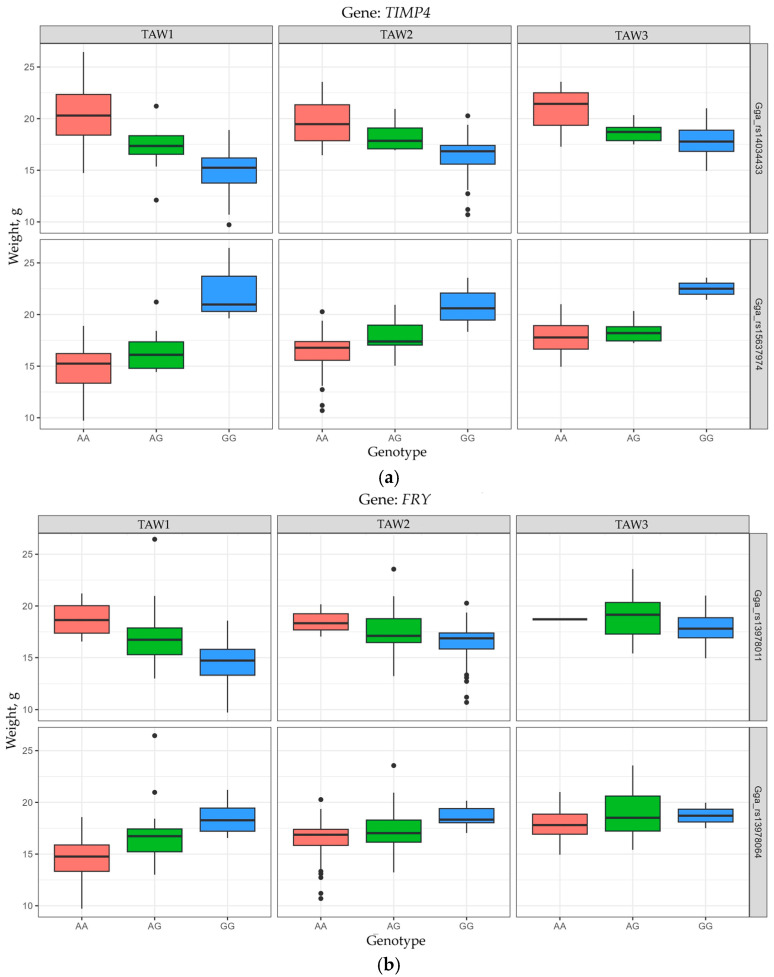
Data visualization box plots (using ggplot2) for thick albumen weight (TAW) of eggs in F_2_ hens of the resource population depending on the genotypes for the *TIMP4* (**a**) and *FRY* (**b**) genes: TAW1, TAW2, and TAW3 correspond to TAW values at the ages of 18–28, 29–41, and 42–52 weeks, respectively.

Significant correlations with higher TAW values (*p* < 0.001) were established for the AA genotype at the loci Gga_rs14034433 (12:5165421; *TIMP4*) and Gga_rs13978011 (1:175956011; *FRY*) and for the GG genotype at the loci Gga_rs15637974 (12:5176426; *TIMP4*) and Gga_rs13978064 (1:175971650; *FRY*). Moreover, the established significant effects of allelic variants of the *TIMP4* gene on TAW were noted in all studied periods of egg laying, while these effects of the *FRY* gene were found for TAW1 and TAW2 (Figure 4a,b and Supplementary Table S3).

## 4. Discussion

The advent of genome mapping and genomics in domestic animals, including poultry, has opened up unprecedented opportunities for pinpointing molecular markers and genes related to performance traits. Hence, it provides invaluable information for genetic monitoring and breeding [147,148,149,150,151], and the GWAS approach has proven instrumental in inferring the potential associations between markers/genes and economically important traits [152,153,154,155]. In this study, we explored and established the associations of SNPs and candidate genes in relation to the weight characteristics of the main components of the egg, i.e., yolk, albumen, and shell, in F_2_ resource population hens. F_2_ resource populations, derived from crossing phenotypically and genetically divergent parental forms are a classic and highly effective model for the initial identification of genomic regions and specific genetic variants associated with target traits [112,115,156]. This approach allows for the expansion of genetic variability and increases the power of analysis to detect loci that may have low frequencies or not exhibit significant polymorphism within a single breed. In F_2_ resource populations, segregation occurs at many loci, enhancing contrast for the studied traits [112,115,156].

In this study, an F_2_ resource population was obtained using two breeds, RW and CW, contrastingly selected for egg productivity traits, including EW. To search for genome-wide associations for weight parameters of the main egg components, 142 F_2_ individuals with contrasting phenotypes for EW were selected, taking into account their origin. Analysis of phenotypic variability in this population revealed a significant effect of the age-at-lay factor (*p* < 0.001) on the main egg components, in particular, EW (*F* = 237.9), YW (*F* = 245.5) and TAW (*F* = 40.3). The influence of the parental group factor on the studied traits in the age-related dynamics of egg production was also established. The hatching factor (hatch batch), however, did not have a significant effect on the weight parameters of egg components in hens of the studied resource population.

Our study examined the weight parameters of egg components (yolk, albumen, thick albumen, and shell) at three different laying stages. The majority of significant associations were found in the first recorded laying period, between 18 and 28 weeks of age, for yolk weight (YW1) and albumen weight (TAW1), suggesting a more pronounced influence of genetic factors at the onset of egg production.

In addition to the established significant associations for YW1 and TAW1, our GWAS also revealed significant SNPs in the initial period of egg laying for ESW1, as well as for TAW2 in the age range from 29 to 42 weeks. Herewith, seven PCGs were established that were associated with YW1 (*SYTL5*, *ALDH1A3*, *GABRG3*, *VCL,* and *HYDIN*) and TAW1 (*FRY*, *GABRG3,* and *TIMP4*). An additional examination of allelic variants at the loci of *HYDIN*, *VCL*, *ALDH1A3*, *TIMP4,* and *FRY* showed that individual genotypes at these loci significantly correlated with higher values of the YW1 and TAW1 traits (*p* < 0.001).

Other authors also demonstrated the associations of PCGs that we identified with egg production indicators, egg quality and growth indicators in chickens and other farm animals. For instance, Sun et al. [157] established significant associations of the *HYDIN* gene with EW in ducks, which is consistent with our results. The yolk is the second major component of the egg after the albumen and also directly influences egg weight, although to a lesser extent than the albumen [82]. It has been shown that egg weight was positively correlated with yolk weight [83]. Other studies found an association of *HYDIN* with egg production and the development of the reproductive system in chickens [158]. They also found associations with meat quality, e.g., the metabolism of glycerophospholipids in the breast muscle of chickens at the age of 42 and 126 days [159]. The egg yolk is a derivative of ovarian follicles, so the intensity of development and the weight of the follicles are directly related to YW [160,161,162]. Moreover, glycerophospholipids are part of the yolk and determine its nutritional value [163,164,165,166].

The expression of three genes identified in this study, i.e., *GABRG3*, *TIMP4,* and *VCL*, has been previously studied in the cells of the reproductive organs and oviduct of laying hens in relation to the egg laying physiology and egg performance. Specifically, Yan et al. [167] reported differences in the expression level of the *GABRG3* gene in ovarian stromal cells and F5 follicle membranes in ducks with high and low egg production. This is also relevant to our data regarding the effect of *GABRG3* on YW of the egg. Du et al. [168] observed different levels of the *TIMP4* gene expression in the magnum fibroblasts of the oviduct in ducks depending on the period of laying and its absence. In a study by Yang et al. [169], upregulation of the *VCL* gene was established in the eggshell glands of hens that had a higher egg productivity. Considering that egg protein is secreted in the magnum section of the oviduct, the results of the above studies can indirectly be linked to our experimental data for the effect of *TIMP4* on TAW in the studied chicken population.

An association with the qualitative eggshell characteristics in chickens has also been shown for the *FRY* gene. That is, the *FRY* gene was associated with the eggshell strength in 36-week-old hens [170]. The shell strength depends to some extent on its thickness [35,171,172,173], which affects both ESW and EW [174].

A number of investigations have also established an association between the mentioned *VCL*, *SYTL5*, *FRY,* and *TIMP4* genes and indicators characterizing and associated with BW and growth in poultry. Specifically, the *VCL* gene is involved in the differentiation and development of muscle tissue [175], pectoral muscles [176], and the development of wooden breast myopathy in broilers [177]. *SYTL5* has been linked to breast weight [178], whereas BW at 35 days of age was presumably affected by *FRY* [179]. *TIMP4* was relevant to BW at 6 and 9 weeks of age [180]. In a study by Wolc et al. [83], a positive correlation was shown between EW and BW of laying hens. The association of the *HYDIN*, *ALDH1A3,* and *GABRG3* genes with growth and development indicators has also been shown in other species of farm animals, in particular, sheep [181,182] and cattle [183]. It should also be noted that a number of studies have demonstrated a relation between the *ALDH1A3* gene and amino acid metabolism in the liver of growing laying hens [184], feather pigmentation in ducks [185], and yellow pigment deposition in chicken skin [186].

Collectively, the available findings from other studies are consistent with our data with respect to the suggested influence of the *HYDIN*, *GABRG3*, *TIMP4*, *FRY,* and *VCL* genes on the egg production and egg quality parameters in laying hens. For other genes identified in our work, certain studies have shown their association with growth parameters in hens (*SYTL5*) and pigmentation in duck feathers and chicken skin (*ALDH1A3*). According to some investigations [187], there is a certain overlap of QTLs for growth and egg production traits. For example, chicken breast development may be a potentially correlated and selected trait for egg performance [188,189]. The SNPs and PCGs identified in our study can be considered prospective genetic markers associated with weight parameters of egg components in laying hens. Assessing the potential of using these genetic markers and their inclusion in DNA marker panels for genomic and marker-assisted selection of chickens, including specific breeds and purebreds, requires further studies. The latter should be aimed at validating the obtained data in independent populations using GWAS approaches, as well as whole-genome genotyping and sequencing to confirm and more thoroughly examine the association between the PCGs identified in this study and egg quality parameters in laying hens. Further studies using GWAS approaches and whole-genome genotyping and sequencing are needed to confirm and explore in more detail the association of these PCGs with egg quality parameters in layers.

## 5. Conclusions

Using the Illumina Chicken 60K SNP iSelect BeadChip, we conducted a GWAS on F_2_ resource population layers for traits characterizing the weight parameters of the main egg components, i.e., yolk, albumen, and shell. As a result, 122 SNPs and 53 candidate genes (in SNP positions) were identified as having a highly significant association with the studied parameters. These included YW1 (33 SNPs, 17 genes), TAW1 and TAW2 (87 SNPs, 35 genes), and ESW1 (4 SNPs, 3 genes). The maximum numbers of identified SNPs and candidate genes were observed on GGA1 (29 SNPs, 14 genes) and GGA2 (14 SNPs, 6 genes). Seven PCGs were localized in the regions of 2 to 4 SNPs associated with the studied traits, including *SYTL5* (YW1, TAW1), *FRY* (TAW1), *GABRG3* (YW1, TAW1), *ALDH1A3* (YW1), *VCL* (YW1), *HYDIN* (YW1) and *TIMP4* (TAW1). Significant associations with high values of the YW1 trait were revealed for individual genotypes at the *HYDIN*, *VCL,* and *ALDH1A3* loci. Also, effects of individual genotypes at the *TIMP4*, *FRY*, *TIMP4,* and *FRY* loci on higher TAW1 values were shown.

The experimental data produced are of great importance for understanding the molecular genetic basis for the formation and implementation of the productive potential in hens, including the weight parameters of the egg and its main components (yolk, albumen, and shell). The identified SNPs and PCGs require further investigation and can be used as potential genetic markers in layer breeding programs aimed at improving egg quality indicators. In particular, they can be instrumental for predicting the genetic potential of egg productivity and weight parameters of eggs and their main components in hens at an early age and for targeted selection of layers with high genetically determined potential for egg performance and EW.

## Figures and Tables

**Table 1:** Weight indices of eggs and their components in F_2_ hens of the resource population.

Age, Weeks	Indicator ^1^	Weight, g				
		Egg	Yolk	Egg White	Thick Albumen	Shell
18–28	M ± m	43.00 ± 0.36	12.00 ± 0.20	24.90 ± 0.38	15.30 ± 0.26	6.10± 0.11
	Min...Max	33.5...57.8	9.9...15.3	21.5...34.9	9.7...20.5	4.7...8.4
	CV, %	8.2	11.2	10.6	16.3	13.3
29–41	M ± m	50.80 ± 0.54 *	15.60 ± 0.17 *	28.70 ± 0.30 *	16.80 ± 0.25 *	6.50 ± 0.08 *
	Min...Max	36.6...63.7	12.5...19.2	26.0...35.6	10.7...23.5	5.1...8.1
	CV, %	9.3	7.7	7.3	12.6	8.2
42–52	M ± m	57.80 ± 0.53 *	18.70 ± 0.21 *	31.70 ± 0.33 *	18.10 ± 0.27 *	7.40 ± 0.12 *
	Min...Max	51.4...70.0	15.4...20.6	24.2...36.5	14.9...23.6	5.9...9.8
	CV, %	6.7	7.9	7.6	10.5	12.1

^1^ M ± m, mean, and standard error of the mean; Min...Max, minimum and maximum values; CV, coefficient of variation. * The differences relative to the same indicator of the previous period are significant at *p* < 0.01.

**Table 2:** Results of decomposition of fixed factors by significance level of their effects on phenotypic indices of egg components in F_2_ resource population hens.

Traits	Effects ^1^						*R* ^2^
	Age		Parent Group		Hatch		
	*F*	*p*-Value	*F*	*p*-Value	*F*	*p*-Value	
Egg weight	237.9	0.000 ***	1.66	0.119	2.02	0.077 ^t^	0.717
Yolk weight	245.5	0.000 ***	1.16	0.330	1.92	0.109	0.762
Egg white weight	94.5	0.000 ***	2.07	0.058 ^t^	2.23	0.067 ^t^	0.584
Thick albumen weight	40.3	0.000 ***	2.19	0.046 *	1.58	0.181	0.469
Eggshell weight	51.4	0.000 ***	0.49	0.814	0.91	0.458	0.492

^1^ *F*, Fisher’s *F*-statistics; *R*^2^, coefficient of determination of the model by a trait. Significant effects: * *p* < 0.05; *** *p* < 0.001; ^t^ tendency (*p* < 0.10).

**Table 3:** Values of least squares (LS means)-based estimates for the phenotypic egg component parameters in the F_2_ resource population, taking into account the age of egg laying and using a set of fixed factors (parent family groups and incubation batches).

Traits	LS Means-Based Trait Estimates		
	Age, Weeks		
	18–28	29–41	42–52
Egg weight, g	44.05 ± 0.69	52.00 ± 0.70	57.74 ± 0.78
Yolk weight, g	12.21 ± 0.30	15.97 ± 0.30	18.64 ± 0.33
Egg white weight, g	25.65 ± 0.45	29.43 ± 0.44	31.45 ± 0.49
Thick albumen weight, g	15.28 ± 0.34	16.90 ± 0.34	18.27 ± 0.37
Eggshell weight, g	6.03 ± 0.13	6.69 ± 0.13	7.35 ± 0.14

**Table 4:** Distribution of significant (at *p* < 1.06 × 10^−6^) single nucleotide polymorphisms (SNPs) associated with the respective indicators of the main egg components in F_2_ resource population hens along chicken chromosomes (GGA).

Trait	No. of SNPs	Chromosomes
Thick albumen weight (18–28 weeks)	85	GGA1–GGA5, GGA7–GGA9, GGA11, GGA12, GGA14–GGA18, GGA20, GGA25, GGA27
Thick albumen weight (29–41 weeks)	2	GGA2, GGA8
Yolk weight (18–28 weeks)	33	GGA1, GGA5, GGA6, GGA7, GGA9–GGA11, GGA17, GGA20, GGA21
Shell weight (18–28 weeks)	4	GGA1, GGA3, GGA17

**Table 5:** Priority candidate genes associated with weight parameters of the main egg components in F_2_ hens of the resource population.

Chromosomes ^1^	Genes	SNPs ^2^	SNP Position	*β*	*R* ^2^	*p*-Value	Trait ^3^
GGA1	*SYTL5*	GGaluGA038925	114,366,975	2.158	0.2862	7.91 × 10^−8^	YW1
		GGaluGA038927	114,379,202	5.183	0.3453	3.05 × 10^−7^	TAW1
	*FRY*	Gga_rs13978011	175,956,011	2.310	0.2570	3.98 × 10^−7^	TAW1
		Gga_rs13978064	175,971,650	2.137	0.2584	3.13 × 10^−7^	TAW1
	*GABRG3*	Gga_rs13939653	132,426,482	2.667	0.3725	3.98 × 10^−8^	TAW1
		Gga_rs15424427	132,514,348	6.371	0.2772	6.67 × 10^−8^	TAW1
		Gga_rs13939653	132,426,482	5.064	0.2163	5.52 × 10^−7^	YW1
GGA6	*VCL*	Gga_rs16546266	16,277,262	−1.609	0.2653	2.41 × 10^−7^	YW1
		Gga_rs14576710	16,283,091	−1.608	0.2646	2.15 × 10^−7^	YW1
GGA10	*ALDH1A3*	Gga_rs14952507	17,878,734	1.754	0.2295	5.60 × 10^−7^	YW1
		Gga_rs14952510	17,878,899	1.754	0.2295	5.60 × 10^−7^	YW1
		GGaluGA072046	17,898,445	1.784	0.2450	1.48 × 10^−7^	YW1
		Gga_rs10730304	17,910,258	1.747	0.2343	3.35 × 10^−7^	YW1
GGA11	*HYDIN*	Gga_rs15601378	1,592,394	1.819	0.2504	9.87 × 10^−7^	YW1
		Gga_rs14018273	1,614,369	1.846	0.2901	9.43 × 10^−7^	YW1
		GGaluGA074476	1,626,527	1.929	0.2836	1.85 × 10^−7^	YW1
GGA12	*TIMP4*	Gga_rs14034433	5,165,421	2.628	0.2611	2.67 × 10^−7^	TAW1
		Gga_rs15637974	5,176,426	2.664	0.2802	8.12 × 10^−8^	TAW1

^1^ GGA, *Gallus gallus* chromosome. ^2^ Single nucleotide polymorphisms (SNPs). ^3^ YW1, yolk weight at 18–28 weeks; TAW1, thick albumen weight at 18–28 weeks of age; *β*, regression coefficient for quantitative traits; *R*^2^, regression *R*-squared showing proportion of variance explained by SNP.

## Data Availability

The genotyping data presented in this study can be shared with third parties upon approval by the GWMAS Consortium. Other original contributions presented in the study are included in the article and Supplementary Materials; further inquiries can be directed to the corresponding authors, with permission provided by the chickens’ owners.

## Supplementary Materials

The following supporting information can be downloaded at https://www.mdpi.com/article/10.3390/ani15233391/s1, Table S1: SNPs and candidate genes associated with the weight of main egg components in F_2_ resource population hens at different egg lay periods; Table S2: Gene ontology (GO) term enrichment analysis at the positions of the determined SNPs in F_2_ chickens of the resource population; Table S3: Egg weight components in F_2_ hens of the resource population depending on the genotype for the genes *ALDH1A3*, *HYDIN*, *VCL*, *FRY* and *TIMP4*.
