# Supplementary material for: Genome-Wide Egg Hunt: Unhiding Candidate Genes for Egg Component Traits in Layers of an F2 Resource Population
Source: Animals (Basel). 2025 Nov 24;15(23):3391. doi: 10.3390/ani15233391 (PMC12691444; doi:10.3390/ani15233391)
Supplement: Supplementary file 1 [file animals-15-03391-s001.zip › Supplementary Table S3.pdf]

Table S3. Egg weight components in F_2 hens of the resource population depending on the genotype for the genes *ALDH1A3*, *HYDIN*, *VCL*, *FRY* and *TIMP4*.

Yolk weight						
**Gene**	**SNP (chromosome: position)**	**Genotype**	***n***	**YW1**	**YW2**	**YW3**
ALDH1A3	Gga_rs10730304 (10: 17910258)	G/G	13	14.95 ± 0.71 ac***, bc*	16.3 ± 0.44	18.52 ± 0.52
		G/A	41	13.22 ± 0.34 ab*, bc*	15.94 ± 0.28	18.54 ± 0.21
		A/A	88	11.46 ± 0.22 ac***, ab***	15.41 ± 0.17	18.11 ± 0.17
	Gga_rs14952507 (10: 17878734)	A/A	13	15.28 ± 0.56 ad*, ae***	16.02 ± 0.51	18.74 ± 0.52
		A/C	28	13.23 ± 0.49 ad*, de***	15.96 ± 0.34	18.62 ± 0.25
		C/C	101	11.64 ± 0.2 ae***, de**	15.55 ± 0.16	18.07 ± 0.16
	Gga_rs14952510 (11: 17878899)	A/A	13	15.28 ± 0.56 ab*, ac***	16.02 ± 0.51	18.74 ± 0.52
		A/G	28	13.23 ± 0.49 ab*, bc***	15.96 ± 0.34	18.62 ± 0.25
		G/G	101	11.64 ± 0.2 ac***, bc**	15.55 ± 0.16	18.07 ± 0.16
	GGaluGA072046 (10: 17898445)	A/A	13	15.28 ± 0.56 ab**, ac***	16.02 ± 0.51	18.74 ± 0.52
		A/G	39	13.09 ± 0.35 ab**, bc***	16.02 ± 0.28	18.67 ± 0.21
		G/G	90	11.5 ± 0.22 ac***, bc***	15.45 ± 0.16	18.01 ± 0.17
HYDIN	Gga_rs15601378 (11: 1592394)	G/G	13	15.01 ± 0.87 ac***	16.46 ± 0.50 ac**	18.47 ± 0.33
		G/A	34	13.47 ± 0.36 ab***	16.29 ± 0.23 ab**	18.56 ± 0.24
		A/A	96	11.51 ± 0.2 ac***, ab***	15.27 ± 0.17 ab**, ac**	18.11 ± 0.17
	Gga_rs14018273 (11: 1614369)	A/A	16	15.35 ± 0.75 ac***, ab**	16,52 ± 0.46 ac**	18.19 ± 0.32
		A/G	35	13.04 ± 0.29 ab**, bc***	16.16 ± 0.24 bc**	18.58 ± 0.24
		G/G	92	11.46 ± 0.21 ac***, bc***	15.28 ± 0,17 ac**, bc**	18.08 ± 0.17
	GGaluGA074476 (11: 1626527)	G/G	13	15.06 ± 0.92 ac***	16.52 ± 0.57 ac*	18.12 ± 0.39
		G/A	35	13.58 ± 0.35 ab***	16.31 ± 0.23 ab**	18.73 ± 0.21
		A/A	95	11.43 ± 0.19 ac***, ab***	15.26 ± 0.17 ac*, ab***	18.02 ± 0.17
VCL	Gga_rs16546266 (6: 16277262)	A/A	29	10.72 ± 0.41 ac***, ab*	15.44 ± 0.39	18.41 ± 0.29
		A/G	54	11.54 ± 0.23 ab*, bc***	15.44 ± 0.18	18.08 ± 0.22

		G/G	59	13.74 ± 0.3<br>ac***, bc***	15.95 ± 0.22	18.39 ± 0.19
	Gga_rs14576710 (6: 16283091)	G/G	28	10.72 ± 0.41<br>ac***, bc*	15.44 ± 0.39	18.4 ± 0.29
		G/A	55	11.54 ± 0.22<br>bc*, ab***	15.34 ± 0.19	18.08 ± 0.22
		A/A	58	13.73 ± 0.3<br>ac***, ab***	16.06 ± 0.21	18.31 ± 0.19
**Thick albumen weight**						
**Gene**	**SNP (chromosome: position)**	**Genotype**	***n***	**TAW1**	**TAW2**	**TAW3**
FRY	Gga_rs13978064 (1: 175971650)	G/G	9	18.52 ± 0.61<br>ca***, bc*	18.59 ± 0.42<br>ca***, cb*	18.73 ± 0.42<br>ca*
		G/A	27	17.05 ± 0.62<br>ab***, bc*	17.4 ± 0.53<br>ba*	19.01 ± 0.52<br>ba**
		A/A	106	14.59 ± 0.19<br>ac***, ab***	16.4 ± 0.19<br>ac*, ab*	17.87 ± 0.17<br>ab**, ac*
	Gga_rs13978011 (1: 175956011)	A/A	6	18.76 ± 0.89<br>ac***, ab*	18.51 ± 0.68<br>ac**	18.71 ± 0
		A/G	30	17.1 ± 0.54<br>ab*, bc***	17.52 ± 0.49<br>bc*	19.12 ± 0.46<br>bc*
		G/G	105	14.57 ± 0.19<br>ac***, bc***	16.4 ± 0.19<br>ca**, cb*	17.87 ± 0.17
TIMP4	Gga_rs15637974 (12: 5176426)	G/G	5	22.35 ± 1.87<br>ac***, bc*	20.83 ± 1.36<br>ca**, cb*	22.5 ± 0.78<br>ca***, cb***
		G/A	24	16.43 ± 0.4<br>ab**, bc*	17.88 ± 0.34<br>ba***, bc**	18.33 ± 0.23<br>ba*, bc***
		A/A	114	14.79 ± 0.19<br>ac***, ab**	16.29 ± 0.18<br>ac**, ab***	17.84 ± 0.17<br>ac***, ab*
	Gga_rs14034433 (12: 5165421)	A/A	6	20.44 ± 2.09<br>ac**	19.74 ± 1.33<br>ac**	20.76 ± 1.39<br>ac*
		A/G	13	17.13 ± 0.77<br>bc**	18.3 ± 0.47<br>bc**	18.71 ± 0.33
		G/G	123	14.87 ± 0.18<br>ac**, bc**	16.4 ± 0.18<br>ca**, cb**	17.84 ± 0.16<br>ca*, cb**

Note: YW1, yolk weight at 18–28 weeks of age; YW2, same at 29–41 weeks of age; YW3, same at 42–52 weeks of age; TAW1, thick albumen at 18–28 weeks of age; TAW2, same at 29–41 weeks of age; TAW3, same at 42–52 weeks of age. Significance notation: a, AA; b, AG; c, GG; d, AC; e, CC; *, $p < 0.05$; **, $p < 0.01$; ***, $p < 0.001$.
